# Geographic Distribution of HCV Genotypes and Efficacy of Direct-Acting Antivirals in Chronic HCV-Infected Patients in North and Northeast China: A Real-World Multicenter Study

**DOI:** 10.1155/2022/7395506

**Published:** 2022-04-29

**Authors:** Wencong Li, Jing Liang, Jihong An, Lingdi Liu, Yihui Hou, Lu Li, Wen Zhao, Luyao Cui, Ningning Xue, Zaid Al-Dhamin, Tao Han, Yuemin Nan, Liaoyun Zhang

**Affiliations:** ^1^Department of Traditional and Western Medical Hepatology, Third Hospital of Hebei Medical University, Hebei Key Laboratory of Mechanism of Liver Fibrosis in Chronic Liver Disease, Shijiazhuang 050051, China; ^2^Department of Hepatology, Tianjin Third Central Hospital, The Third Central Clinical College of Tianjin Medical University, Tianjin 300170, China; ^3^Department of Infectious Diseases, Inner Mongolia People's Hospital, Hohhot 010017, China; ^4^Department of Infectious Diseases, The First Affiliated Hospital of Shanxi Medical University, Taiyuan 030001, China

## Abstract

**Objective:**

To assess the geographic distribution of HCV genotypes, effectiveness, and safety of DAA treatment for HCV-infected patients in North and Northeast China.

**Methods:**

The geographic distribution of HCV genotypes was analyzed in 2162 patients recruited from April 2018 to February 2021. Sustained virologic response rates at 12 (SVR12) or 24 (SVR24) weeks posttreatment and safety were analyzed in 405 patients who completed DAA treatment according to patient baseline characteristics and treatment.

**Results:**

Four genotypes and six subtypes were identified as follows: 1b (1187, 54.90%), 2a (790, 36.54%), 3a/b (134, 6.20%), 6a/n (44, 2.04%), mixed genotypes (2a-6a or 2a-3a) (7, 0.32%). Overall, 99.01% patients achieved SVR12, while 98.43% achieved SVR24. All patients treated with elbasvir/grazoprevir (EBR/GZR), sofosbuvir/velpatasvir ± ribavirin (SOF/VEL ± RBV), and SOF/ledipasvir (LDV) achieved SVR12 or SVR24; 92.86% SVR12 and 95.83% SVR24 were observed in patients using SOF + RBV. SVR12 was higher in noncirrhosis versus compensated cirrhosis patients (100% vs. 97.09%, *p*=0.022). No severe drug-related adverse event was observed.

**Conclusions:**

Genotypes 1b and 2a were dominant subtypes in North and Northeast China. The approved drug regimens EBR/GZR and SOF/LDV for subtype 1b and SOF/VEL for nongenotype 1b are the optimal effective and safety profile.

## 1. Introduction

Hepatitis C virus (HCV) infection is globally estimated at more than 185 million, of which 350000 cases died from HCV infection each year [1]. HCV infection is the second common chronic viral hepatitis and approximately 10 million people living with the disease in China [2]. The majority of patients have a long duration of HCV infection from earlier exposure as blood donors or users of blood products from the late 1980s and early 1990s in North and Northeast China. Therefore, most patients are over 50 years old, and the incidence of HCV-related liver cirrhosis and hepatocellular carcinoma (HCC) are gradually increased. The antiviral therapies could reduce the risk of the disease progression and mortality caused by liver decompensation and HCC.

Recently, direct-acting antivirals (DAAs) therapy for HCV facilitates the eradicating of viral hepatitis by 2030. The latest pan-genotypic regimens were recommended in the new guidelines [3–5], which can induce excellent sustained virologic response (SVR) rates independent of HCV genotype. However, pan-genotypic DAA regimens are still limited according to different reimbursement criteria and treatment policies established by local governments and agencies. In China, three DAA regimens, elbasvir/grazoprevir (EBR/GZR), ledipasvir/sofosbuvir (LDV/SOF), and sofosbuvir/velpatasvir (SOF/VEL), have been approved based on the genotypes by the National Healthcare Security Administration in December 2019. Thus, knowledge on the HCV genotype is now critical for tailoring DAA therapy [2]. Furthermore, it is conducive to analyze the HCV genotype distribution for the prevention and antiviral treatment of HCV infection.

HCV can be classified into seven major genotypes and 67 confirmed subtypes according to genomic heterogeneity [6]. HCV genotypes 1–6 have been found in China, of which 1b (54.90%) and 2a (36.54%) are the two predominant subtypes, especially in North and Northeast China [7]. It has been demonstrated that HCV genotype distribution varies by transmission mode. Patients with genotype 1 or 2 are mostly associated with blood transfusions, while genotypes 3b and 6a were more frequent in intravenous drug users [8, 9]. Such data on the distribution of HCV genotypes and effectiveness and safety of DAAs in real-world clinical practice are essential for guiding patients and physicians in making decisions about treatment regimens, as well as informing health care policy around treatment coverage. The latest data on HCV infection and therapies in those regions are still rare. Therefore, the aim of our study was to assess the geographic and demographic distribution of HCV genotypes and the real-world status, effectiveness, and safety of DAA treatment for chronic HCV-infected patients in North and Northeast China.

## 2. Materials and Methods

### 2.1. Study Population and Design

**Figure 1:**
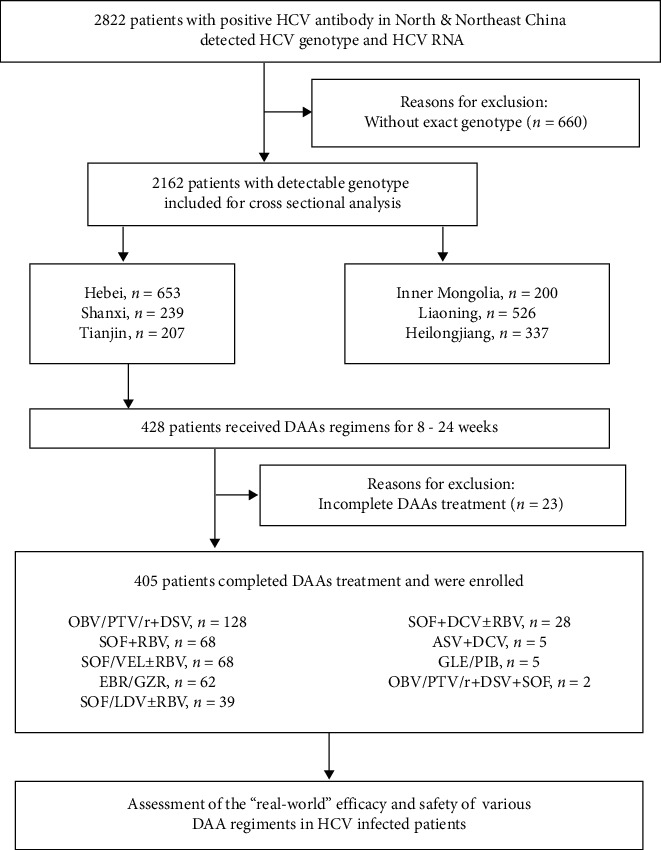
Flowchart of chronic hepatitis C virus (HCV) infected patient enrollment and follow-up. DAAs: direct-acting antivirals; OBV/PTV/r: ombitasvir/paritaprevir/ritonavir; DSV: dasabuvir; SOF: sofosbuvir; RBV: ribavirin; EBR/GZR: elbasvir/grazoprevir; VEL: velpatasvir; DCV: daclatasvir; ASV: asunaprevir; LDV: ledipasvir; and GLE/PIB: glecaprevir/pibrentasvir.

The multicenter cross-sectional observational study enrolled 2822 patients with chronic HCV infection from April 2018 to February 2021 from six provinces/municipalities of North (Hebei, Tianjin, Shanxi, and Inner Mongolia) and Northeast (Liaoning and Heilongjiang) China. Diagnosis of chronic HCV infection was based on China's guideline of prevention and treatment for hepatitis C (2019) [2]. The flowchart of chronic HCV-infected patient enrollment and follow-up was shown in Figure 1. A total of 2162 patients with exact HCV genotype/subtype were recruited. Their demographic and clinical characteristics, including age, gender, regions, HCV RNA, and HCV genotype were documented. Enrollment was stratified according to the population demographics of each region.

In the patients enrolled in the cross-sectional phase of the study, 405 patients received the DAAs treatment depending on the discretion of physicians, and no randomization or protocol-driven treatment was implemented. The patients at least 18 years of age who completed DAAs treatment were recruited for our analysis, no matter treatment-naïve or not. Alcohol intake is an independent predictor of cirrhosis in subjects with chronic HCV infection and an independent predictor of death in subjects with HCV infection as previously reported [10], while no patient in our study suffered alcoholic disease according to the ACG Clinical Guideline [11]. After withdrawal, the patients were visited every 12 weeks. For baseline and every visit, we collected the demographic information, biochemistry, hematology, blood coagulation functions, HCV RNA loads, and adverse events (AEs).

### 2.2. Ethics Approval and Consent to Participate

The study was reviewed and approved by the Human Ethics Committee, Third Hospital of Hebei Medical University (K2019-014-2), and by the local research ethics committee at each centre, in accordance with the Declaration of Helsinki. It was registered in Chinese Clinical Trials Registry (no. ChiCTR2000031821). All patients provided written informed consent.

### 2.3. Detection of Antibody, Viral Load, and Genotypes of HCV

Serum HCV antibody was detected by enzyme-linked immunosorbent assay (ELISA) using a commercial detection kit (Livzon Diagnostics Inc., Zhuhai, China). The assay of plasma HCV RNA loads and the HCV genotypes were performed in Dian Diagnostics Technology Limited Company by polymerase chain reaction (PCR)-fluorescence probing using HCV RNA Quantitative Fluorescence Diagnostic Kit (Sansure Biotech Inc., Ltd., Hunan, China) and PCR-sequencing analysis using Diagnostic Kit for HCV Genotyping (Daan Gene Co., Ltd. of Sun Yat-Sen University, Guangdong, China). The lowest detection limit of HCV RNA is 25 IU/ml.

### 2.4. Study Assessment

Liver cirrhosis was diagnosed based on clinical evidence of cirrhosis (e.g., liver nodularity and/or splenomegaly on imaging, platelet count <150,000/mm^3^, etc.), or FibroScan and FibroTouch detecting liver stiffness measurement more than 12.5 kPa, and/or liver biopsy showing cirrhosis, if biopsy results were available. Decompensated cirrhosis was defined as cirrhosis with sequelae such as ascites, hepatic encephalopathy, variceal hemorrhage, or Child–Turcotte–Pugh score ≥7 [3].

Virological response at the end of treatment (EOT) was defined as undetectable HCV RNA at EOT; SVR was defined as undetectable HCV RNA at 12 (SVR12) or 24 (SVR24) weeks posttreatment.

### 2.5. Statistical Analysis

Continuous variables are expressed as median (interquartile range, IQR) or mean ± standard deviation and categorical variables as frequencies or percentages (%) of patients. All data were analyzed using the Statistical Package for the Social Sciences (SPSS) Version 21.0 (IBM Corp., Armonk, NY, USA). Kruskal–Wallis *H* test was performed for quantitative data to compare the differences among more than two groups. Proportions were compared using Chi-squared test and Fisher's exact test where numbers were small. Ordinal categorical variables were tested using Mann–Whitney or Kruskal–Wallis test. The *p* values <0.05 are considered significant.

## 3. Results

### 3.1. Demographic and Virological Characteristics of HCV-Infected Patients in North and Northeast China

The ratio of the HCV patients, as shown in Table 1, was highest in Hebei (653, 30.20%) and Liaoning (526, 24.33%) provinces, followed by Heilongjiang (337, 15.59%), Shanxi (239, 11.05%), Tianjin (207, 9.57%), and Inner Mongolia (200, 9.25%). The median age of patients in each province was Tianjin [58 (51.25, 66)], Hebei [56 (49, 64)], Heilongjiang [55 (48, 64)], Liaoning [55 (49, 63)], Shanxi [53 (44, 60)], and Inner Mongolia [52 (42.25, 60)], respectively. Among 1933 HCV-infected patients with gender records, 1011 (52.30%) were males, and 922 (47.70%) were females. The median HCV RNA loads were in decreasing order as follows: Tianjin > Shanxi > Liaoning > Hebei > Inner Mongolia > Heilongjiang (*p* < 0.001); they were 6.23 (5.51, 6.73), 6.15 (5.54, 6.64), 5.96 (5.18, 6.45), 5.92 (5.05, 6.47), 5.80 (5.55, 6.67), 5.75 (5.06, 6.38) lg·IU/ml, respectively. Of which, Shanxi and Tianjin showed significant higher median HCV RNA loads compared with Hebei and Heilongjiang (*p* < 0.05).

### 3.2. Geographical Distribution of HCV Genotypes

**Figure 2:**
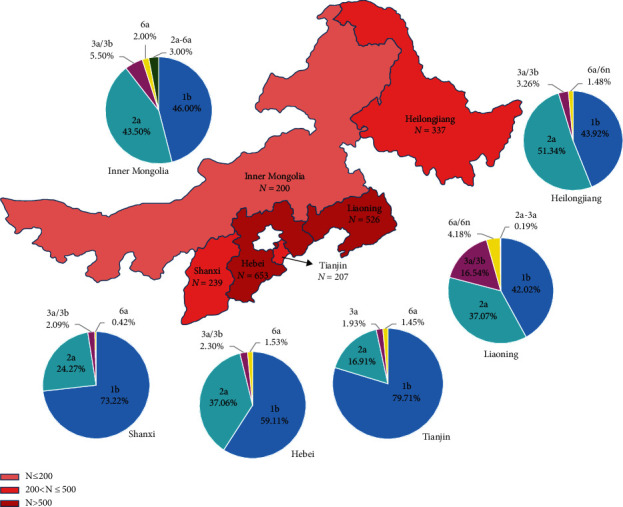
Geographical distribution of HCV genotypes in different provinces/municipalities of North and Northeast China. The numbers of cases in different regions are shown in red on different scales. The numbers and proportions of different HCV genotypes in each region are presented by pie graphs with different colors.

The geographical distribution of HCV genotypes in different provinces of North and Northeast China was shown in Figure 2. There were significant (*p* < 0.05) variations in the distribution of genotypes 1b, 2a, 3, and 6 among patients from all six provinces/municipalities according to the Chi-squared test. Genotypes 1b (1187, 54.90%) and 2a (790, 36.54%) were dominant genotypes from all of the provinces in North and Northeast China, followed by 3a/b (134, 6.20%), 6a/n (44, 2.04%), mixed genotypes (2a-6a or 2a-3a) (7, 0.32%). Genotype 1b was more prevalent in patients from Tianjin (165, 79.71%) and Shanxi (175, 73.22%) than that in those from Liaoning (221, 42.02%) and Heilongjiang (148, 43.92%), while genotype 2a was more prevalent among patients from Heilongjiang (173, 51.34%) than among patients from Shanxi (58, 24.27%) and Tianjin (35, 16.91%). Furthermore, we can see HCV genotype 2a was 7.42% more prevalent than 1b in Heilongjiang, and 87 patients with HCV genotype 3 (*n* = 72 with 3b, *n* = 15 with 3a) were found in Liaoning, which was highly prevalent (16.54%), >3 times more frequent than among patients from other provinces (1.93–5.50%), and no genotype 3b was observed in Tianjin. Genotype 6n HCV infection was lowest of prevalence (<1%) in all regions of Northeast China and not detected in any patients from North China. In addition, there are 6 patients with mixed infections of HCV genotype 2a-6a from Inner Mongolia and 1 patient with HCV genotype 2a-3a infection from Liaoning.

### 3.3. Age-Wise Distribution of Gender and HCV Genotypes in HCV-Infected Patients

To identify the characteristics of HCV-infected patients of different ages, 1474 patients with recorded ages were stratified by ages in 10-year intervals. As displayed in Table 2, HCV was most frequent among individuals aged 56–65 years (31.75%) and 46–55 years (31.41%) followed by those aged 66–75 years (14.38%) (Table 3). With the increase of age, the proportion of men in each age group is gradually decreasing (*p* < 0.001).

Genotype 1b was the most prevalent in all age groups, and no significant difference was found (*p*=0.603). Infection rates of genotype 2a were increased with age (*p* < 0.001), and especially, it was 43.59% in patients over 75 years. Interestingly, neither genotype 3a nor 3b appeared in the groups aged ≤25 and >75 years, and there were only genotype 1b and 2a detected in >75 years old. The mixed genotype infection was mainly found in patients with 36–65 years old.

### 3.4. HCV Viral Load in Different Genotypes and Gender-Wise Genotype Distribution in HCV-Infected Patients

To evaluate the correlation of HCV genotype and HCV viral load, we compared the median HCV RNA loads among different HCV genotypes (Table 3); it was shown that the patients with genotype 2a had significantly lower HCV viral load than that with genotypes 1b and 3 (5.56 lg·IU/ml vs. 6.25 lg·IU/ml and 5.86 lg·IU/ml, respectively, *p* < 0.05). In addition, HCV infection is mainly observed in male patients except for genotype 2a, which was 2.64% more prevalent in females (357, 51.22%) than that in males (340, 48.78%; *p*=0.024). HCV genotype 3 was 38.00% more prevalent in males (69, 69.00%) than that in the females (31, 31.00%; *p*=0.001), and HCV genotype 6 was 44.44% more prevalent in the male (26, 72.22%) than that in the female (10, 27.78%; *p*=0.018). No significant gender differences were observed in genotype 1b and mixed genotypes.

### 3.5. Baseline Characteristics of HCV-Infected Patients Treated with DAAs Regimens

A total of 428 patients received DAAs regimens, of which 405 patients completed DAAs treatment were included in our analysis. The baseline characteristics of those patients were shown in Table 4. 57.28% (232/405) of all patients were females. The median age of the patients was 56 (49, 63) years with a body mass index (BMI) of 24.17 (21.97, 26.30) kg/m^2^. 165 (40.74%) patients were diagnosed as liver cirrhosis at baseline, of which 108 (26.42%) were compensated cirrhosis and 57 (14.07%) decompensated, while 12 (2.96%) patients had a previous history of HCC. For comorbidity, 59 (14.57%) patients with hypertension, 71 (17.53%) with type 2 diabetes, and 3 (0.74%) with hematological system disease were observed. Of 405 patients, 79.26% (*n* = 321) had genotype 1b HCV infection, followed by genotype 2a for 18.02% (*n* = 73), genotype 6a for 1.23% (*n* = 5), genotype 3a for 0.74% (*n* = 3), and genotype 3b for 0.74% (*n* = 3). Most of them (*n* = 378, 93.33%) were treatment-naïve; only 27 (6.67%) patients had a history of prior treatment failure. At baseline, median HCV RNA load in a total 405 patients was 6.23 (5.52, 6.71) lg·IU/ml and was 6.24 (5.52, 6.76), 6.26 (5.53, 6.62), and 5.86 (5.12, 6.49) lg·IU/ml in noncirrhotic, compensated, and decompensated cirrhotic patients, respectively. There are no significant differences of baseline HCV RNA levels among noncirrhotic, compensated, and decompensated cirrhotic patients.

### 3.6. Real-World Effectiveness of Available DAA Therapies for HCV-Infected Patients

**Figure 3:**
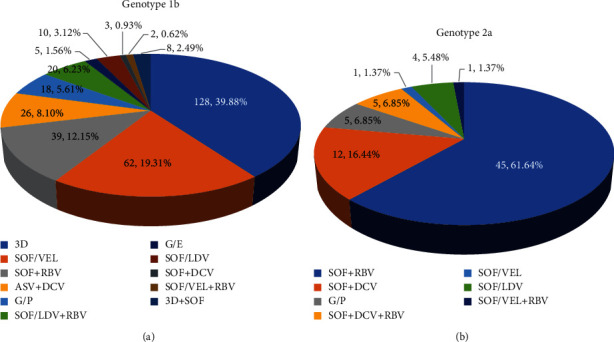
DAAs regimens for HCV genotype 1b and 2a in the real-world study. The number and proportion of different DAAs regimens for HCV genotype 1b (a) and 2a (b) are presented by different colors in the graphs. DAAs: direct-acting antivirals; OBV/PTV/r: ombitasvir/paritaprevir/ritonavir; DSV: dasabuvir; SOF: sofosbuvir; RBV: ribavirin; EBR/GZR: elbasvir/grazoprevir; VEL: velpatasvir; DCV: daclatasvir; ASV: asunaprevir; LDV: ledipasvir; GLE/PIB: glecaprevir/pibrentasvir; and DNV: danoprevir.

In our study, 405 patients from North China completed the 8–24 weeks treatment course: 205 (50.62%) with the sofosbuvir- (SOF-) based regimens (including SOF + RBV, SOF/LDV ± RBV, SOF/VEL ± RBV, SOF + 3D, and SOF + DCV ± RBV), 128 (31.60%) with ombitasvir/paritaprevir/ritonavir (OBV/PTV/r) + dasabuvir (DSV), 62 (15.31%) with elbasvir/grazoprevir (EBR/GZR), 5 (1.23%) with asunaprevir (ASV) + DCV, 5 (1.23%) with glecaprevir/pibrentasvir (GLE/PIB). HCV genotypes 1b and 2a were predominant in North China, and the DAA regimens were shown in Figure 3. Sofosbuvir- (SOF-) based regimens were the most prescribed treatments for HCV genotype 1b-infected patients and SOF + RBV (*n* = 45, 60.81%) for genotype 2a; those DAA regimens were approved earlier by the China Food and Drug Administration (CFDA).

**Figure 4:**
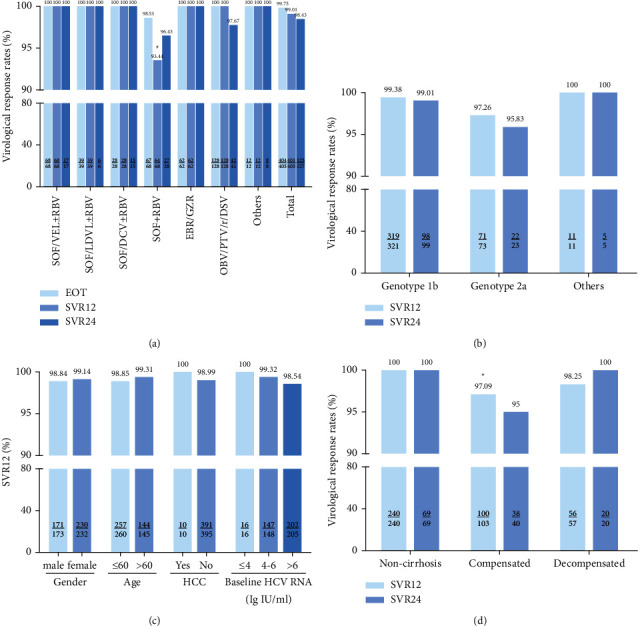
Virological response rates of EOT, SVR12, and SVR24 of DAA therapies in the real-world study. (a) Virological response rates of EOT, SVR12, and SVR24 in patients treated with different DAAs regimens. (b) SVR12 and SVR24 in patients with different HCV genotypes. (c) The SVR12 rates according to the most important baseline and on-treatment features. (d) The rates of SVR12 and SVR24 in patients with noncirrhosis, compensated, and decompensated cirrhosis. Categorical variables were compared using the *χ*^2^ or the Fisher's exact tests. EOT: end of treatment; SVR: sustained virological response. ^#^ SVR12 in OBV/PTV/r + DSV versus SOF + RBV, *p*=0.011, and SVR12 in SOF + RBV versus others, *p*=0.010; ^*∗*^ SVR12 in noncirrhosis versus compensated cirrhosis, *p*=0.022.

The overall virological responses of DAA treatment, as shown in Figure 4, were 99.75% (404/405) at end of treatment, 99.01% (401/405) at 12 weeks posttreatment (SVR12), and 98.43% (125/127) at 24 weeks posttreatment (SVR24) according to per-protocol analysis. All patients treated with DAAs achieved SVR12 except for SOF + RBV (57/61, 93.44%) and SVR24 except for OBV/PTV/r + DSV (42/43, 97.67%) and SOF + RBV (27/28, 96.43%).

Among the predominant HCV genotypes 1b and 2a-infected patients in North China, 319/321 (99.38%) patients with HCV genotype 1b achieved the SVR12 and 100/101 (99.01%) the SVR24, and 71/73 (97.26%) patients with HCV genotype 2a achieved the SVR12 and 23/24 (95.83%) the SVR24, while a small number of patients with other genotypes were detected and all of them achieved the SVR12 and SVR24. There was no significant difference in SVR12/24 among genotypes.

By compared the difference of SVR12/24 among noncirrhosis, compensated cirrhosis, and decompensated cirrhosis, 100% SVR12 (240 patients) and the SVR24 (69 patients) rates were achieved in noncirrhotic patients, while the SVR12 and SVR24 rates were 97.22% (105/108) and 95% (38/40) in the compensated cirrhotic patients and 98.25% (56/57) and 100% (20/20) in the decompensated cirrhotic patients, respectively. There was a significant difference in SVR12 rates between the patients without cirrhosis and with compensated cirrhosis (*p*=0.022).

Moreover, treatment effectiveness was not affected by other baseline and on-treatment features considered, such as gender (*p*=1.000), age >60 years old (*p*=0.624), baseline HCV RNA (≤4 lg IU/ml vs. 4–6 lg·IU/ml vs. >6 lg·IU/ml, *p*=1.000), HCV genotype (*p*=0.208 in SVR12, *p*=0.389 in SVR24), and HCC onset (*p*=1.000).

In this study, 5 (1.23%) patients presented treatment failure. As shown in Table 5, the virological responses of DAA treatment were 100% in CHC and LC group after 2020. Nevertheless, the SVR rate was significant lower in LC group between 2018 and 2019, owing to the unavailability of drugs in patients with liver cirrhosis (the DAA regimens of patients with liver cirrhosis were SOF + RBV and OBV/PTV/r + DSV).

### 3.7. Treatment Safety

Overall, any AEs were reported in 51 (12.56%) patients; most of these were mild to moderate in severity. Hyperbilirubinemia (*n* = 22, 5.42%), anemia (*n* = 12, 2.96%) and fatigue (*n* = 9, 2.22%) were the most frequent and were considered drug-related in 51 patients. Hyperbilirubinemia occurred in 9 (7.03%) of the patients treated with OBV/PTV/r + DSV, 1 (0.78%) of which was discontinued for a while and then completed the treatment. Anemia was more common in patients with SOF + RBV (*n* = 9, 13.24%), which was treated with RBV dose reduction and discontinuation of RBV in 2 (2.94%) and 2 (2.94%) patients, respectively. One patient with hepatocellular carcinoma (HCC) completed SOF + DCV treatment but discontinued for a while due to the recurrence of HCC. And one patient treated with SOF + RBV developed HCC. Other adverse events were detected in less than 1% of patients, such as nausea, dyspepsia, rash, fever, leukopenia, pruritus, dizziness, headache, alopecia, weight loss, amenorrhea, proteinuria, palpitation, and facial edema.

## 4. Discussion

In the current multicenter, cross-sectional observational study, we identified six subtypes (1b, 2a; 3b, 6a; and 3a, 6n) of HCV and two kinds of mixed infection patterns (2a-3a and 2a-6a) in North and Northeast China, with none of genotypes 4 and 5 observed. HCV genotype 1b was the most prevalent genotype in North and Northeast China, accounting for 54.90% of all patients, followed by genotype 2a which contributed to 36.54% of all patients, and other studies had shown the similar results [7, 12]. Regional differences for HCV genotype distribution were also demonstrated. Genotype 1b was detected in a larger proportion in Tianjin (79.71%) and Shanxi (73.22%), while it was decreased in Hebei (59.11%), Inner Mongolia (46%), Liaoning (42.02%), and Heilongjiang (43.92%). Correspondingly, genotype 2a was increased in Hebei (37.06%), Inner Mongolia (43.5%), Liaoning (37.07%), and Heilongjiang (51.34%). Genotype 3 represented 6.27% (132/2105) of the overall infected population and was mainly distributed in Liaoning provinces (*n* = 87), of which the majority of genotype 3 infections were subtype 3b. The proportion of genotypes 3 and 6 gradually increased from North to Northeast China. Genotype 3 and 6 are mainly distributed in Southwest China, like Yunnan, which is mainly related to intravenous drug use [12, 13]. These slight changes may be due to the increasing mobility of the population and different transmission routes.

Mixed infection referred to the detection of more than 2 genetically distinct HCV strains simultaneously [14]. It is well recognized that mixed genotype is more common in patients with repeated HCV exposures [15, 16]. Recently, patients with mixed infection were paid more concern with the development of detection methods [15, 17, 18]. Different patterns of mixed infection had been reported in China, such as 1b/2a, 1b-2k, 6a/2a, 6a/1a, 1b-2k, 3-6, and so on, of which the most common in China was 1b/2a [7, 19]. In our present study, we observed 6 patients with 2a-6a in Inner Mongolia, and only one patient with 2a-3a in Liaoning.

Regarding the association with HCV genotype distribution and demographic characteristics in HCV-infected patients, there is still no complete consensus [7, 20]. In our current study, only 15 (1.06%) patients were observed in the young hepatitis C patients (≤25 years), indicating that hepatitis C protection work has been done adequately and people's awareness of hepatitis C protection has been improved in North and Northeast China. With the increase of age, the proportion of men in each age group was gradually decreasing. Genotypes 1b and 2a were more common in the older group (46–75 years old), while genotypes 3 and 6 were more common in the middle-aged group (36–55 years old). However, no significant correlation between genotype 1b and age was found. In addition, genotype 2a was prominent in females, and other genotypes (1b, 3, 6, and mixed genotypes) were dominant in males. That may be attributed to different habits and behaviors between genders, which can affect the infection routes and transmission outcomes, resulting in different HCV genotypes. Moreover, HCV RNA median level in genotype 1b (6.25 lg·IU/ml) was increased compared to genotypes 2, 3, and 6, indicating that genotype 1b was associated with high HCV viral loads, which is in line with the previous study [7, 20].

It is conducive to analyze the HCV genotype distribution for the prevention and antiviral treatment of HCV infection. Since about 2015, DAAs have had access to the Chinese market, and different pan-genotypic and genotype-specific regimens were available for Chinese patients in recent years. In the current real-world study of chronic HCV-infected patients from North China, we observed 11 regimens for patients with genotype 1b and 7 for genotype 2a. According to the recommendation of China's guideline of prevention and treatment for hepatitis C (2019) [2], GLE/PIB, SOF/VEL, and SOF + DCV were the pan-genotypic regimens for HCV-infected patients regardless of genotype, while OBV/PTV/r + DSV, EBR/GZR, SOF/LDV, and ASV + DCV were effective for genotype 1b and SOF/LDV and SOF + RBV for genotype 2a. Overall, 332/405 (81.98%) patients initiated genotype-specific DAA regimens, only 73/405 (18.02%) received pan-genotypic regimens. Patients with cirrhosis accounted for 40.74%, of which 14.07% were decompensated cirrhosis; and 93.10% of overall patients were treatment-naïve. The available DAA therapies were well tolerated and equally effective overall with a high SVR12 (99.01%) and were consistently greater than 92% for all DAA regimens, regardless of liver status. These data suggest that CHC patients in North China who receive the all-oral DAAs can expect a high cure rate both of treatment naïve and experienced, and patients with cirrhosis, even decompensated cirrhosis, can be treated effectively and safely.

In our study, the commonly used regimens for genotype 1b were OBV/PTV/r + DSV, EBR/GZR, and SOF/VEL, which were gradually approved in China since 2017, while SOF + RBV was used most widely as the earliest genotype-specific regimen recommended for genotype 2a. 100% of patients treated with EBR/GZR, SOF/VEL ± RBV, and SOF/LDV achieved SVR12 or SVR24. Although the patients in our study achieved high rates of SVR12 (99.01%) and SVR24 (98.43%), which is higher than previously reported from other clinical trials [21–23]. In terms of SVR12, SOF + RBV is the least effective, with SVR12 rate of 94.12%, and one patient did not achieve the virological response at EOT. As we all know, SOF + RBV was not recommended to use for genotype 1b patient according to the Chinese guideline; while for genotype 2a, the use of only one kind of DAA drugs (especially for SOF) or combined with RBV also has not been recommended since the pan-genotypic or especial genotypic; single-tablet regimen are available [2]. Since January 1, 2020, three DAA regimens were approved by National Healthcare Security Administration in China, including EBR/GZR (Merck Sharp and Dohme Australia Pty, Ltd.) and SOF + LDV (Gilead Sciences) for HCV genotype 1b, SOF + VEL (Gilead Sciences) for nongenotype 1b-infected patients. The continuous improvement of reimbursement policy will benefit the treatment of HCV patients, helping to achieve the WHO goal of viral hepatitis elimination by 2030.

Further analysis revealed that high SVR rates were observed regardless of gender, age, HCV genotypes, and HCC onset, which was also reported in previous study [24]. Although the baseline HCV RNA values in noncirrhosis [6.24 (5.52, 6.76) lg·IU/ml] were higher compared to those of decompensated cirrhosis [5.86 (5.12, 6.49) lg·IU/ml], additional investigation on the association between SVR and baseline HCV RNA showed no significant difference. The result was not consistent with the real-world study in East Asian hepatitis C patients [25], which displayed that significant independent factors predictive of treatment failure were higher HCV RNA levels. All patients without cirrhosis achieved SVR12 and SVR24, while 105/108 (97.22%) compensated cirrhosis and 56/57 (98.25%) decompensated cirrhosis achieved the SVR12, and 38/40 (95%) and 20/20 (100%) achieved the SVR24, respectively. There was a significant difference only between noncirrhosis and compensated cirrhosis in SVR12. It has been reported that virological failure patients showed more severe liver disease with respect to the SVR [26]. Although patients with cirrhosis achieved SVR12 rate >95%, which is higher than that in the ASTRAL-4 trial (83–94%) [27], we still need to pay more attention to patients with cirrhosis, especially decompensated cirrhosis. Because there were only 5 (1.23%) patients with liver cirrhosis failed treatment in our study, of which 4 patients with genotype 1b or 2a received SOF + RBV, and 1 case with genotype 1b received OBV/PTV/r + DSV, while the regimens of EBR/GZR, SOF/VEL ± RBV, and SOF + DCV showed to be effective in our present study when used in patients with decompensated cirrhosis. In addition, regarding the safety of the DAAs, 51 (12.56%) patients reported not only one AE, most of which were mild to moderate in severity. Hyperbilirubinemia, anemia, and fatigue were the most frequent and were considered drug-related AEs, which did not cause patients to abandon treatment. In our study, 12 (2.96%) patients had a previous history of HCC. In the course of treatment and follow-up, the patient's condition was stable. Only one patient with HCC completed SOF + DCV treatment but discontinued for a while due to the recurrence of HCC, which showed the benefits of DAAs in the recurrence risk of hepatocellular carcinoma. And only one patient treated with SOF + RBV developed HCC. It can be seen that the rising rate of successfully treated HCV patients is favorably impacting the improved outcome of patients with HCV-related chronic liver disease.

In conclusion, the most prevalent HCV genotypes in North and Northeast China patients appear to be 1b and followed in descending order by 2a, 3b, 6a, 3a, and 6n. However, there are significant differences within the region; for example, HCV genotype 2a is more prevalent in patients from Heilongjiang and Inner Mongolia than in those from the other provinces and cities. Genotype 3b was detected commonly in Liaoning. Genotype 1b is associated with the highest viral loads. Notably, the proportion of young patients with hepatitis C is decreasing. The approved drug regimens EBR/GZR and SOF/LDV for subtype 1b and SOF/VEL for nongenotype 1b are the optimal effective and safety profile.

## Figures and Tables

**Table 1:** Demographic and virological characteristics of HCV-infected patients in North and Northeast China.

Area		n (%)	Age median (IQR) (years)	Gender, *n* (%)		HCV RNA median (IQR) (lg·IU/ml)
				Male	Female	
North	Hebei	653 (30.20)	56 (49, 64)	333(56.25)	259 (43.75)	5.92 (5.05, 6.47)
	Shanxi	239 (11.05)	53 (44, 60)	113 (48.50)	120 (51.50)	6.15 (5.54, 6.64)^†^
	Tianjin	207 (9.57)	58 (51.25, 66)	87 (42.03)	120 (57.97)	6.23 (5.51, 6.73)^†^
	Inner Mongolia	200 (9.25)	52 (42.25, 60)	137 (68.50)	63 (31.50)	5.80 (5.55, 6.67)

Northeast	Liaoning	526 (24.33)	55 (49, 63)	188 (55.95)	148 (44.05)	5.96 (5.18, 6.45)^§^
	Heilongjiang	337 (15.59)	55 (48, 64)	131 (42.53)	177 (57.47)	5.75 (5.06, 6.38)^‡§¶^

Total		2162	55 (48, 63)	989 (52.72)	887 (47.28)	5.95 (5.22, 6.52)

HCV: hepatitis C virus; IQR: interquartile range; ^†^*p* < 0.05 versus Hebei; ^‡^*p* < 0.05 versus Shanxi; ^§^*p* < 0.05 versus Tianjin; ^¶^*p* < 0.05 versus Inner Mongolia.

**Table 2:** Age-wise distribution of gender and HCV genotypes in HCV-infected patients.

Age	*n* (%)	Gender, n (%)		HCV genotypes, n (%)				
		Total	Male	1b	2a	3a/b	6a/n	Mixed
≤25	15 (1.02)	15	10 (66.67)	11 (72.33)	3 (20.00)	—	1 (6.67)	—
26–35	127 (8.62)	127	79 (62.20)	92 (72.33)	30 (23.62)	2 (1.57)	3 (2.36)	—
36–45	150 (10.18)	150	91 (60.67)	68 (45.34)	56 (37.33)	17 (11.33)	6 (4.00)	3 (2.00)
46–55	463 (31.41)	463	247 (53.35)	280 (60.48)	142 (30.67)	27 (5.83)	12 (2.59)	2 (0.43)
56–65	468 (31.75)	468	234 (50.00)	282 (60.26)	173 (36.97)	10 (2.14)	2(0.43)	1 (0.21)
66–75	212 (14.38)	212	105 (49.53)	129 (60.85)	80 (37.74)	2 (0.94)	1 (0.47)	—
>75	39 (2.65)	39	17 (43.59)	22 (56.41)	17 (43.59)	—	—	—
Total	1474	1474	783 (53.12)	884 (59.97)	501 (33.99)	58 (3.93)	25 (1.70)	6 (0.41)
*Z*			−3.249	−0.520	−3.400			
*p*-value			0.001	0.603	0.001			

HCV: hepatitis C virus; mixed: mixed infections with two genotypes or subtypes.

**Table 3:** HCV viral load in different genotypes and gender-wise genotype distribution in HCV-infected patients.

Genotypes	HCV RNA Median (IQR) (lg·IU/ml)	Gender, *n* (%)		*χ* ^2^	*p*-value
		Male	Female		
1b	6.25 (5.65, 6.68)	572 (52.28)	522 (47.71)	0.004	0.947
2a	5.56 (4.61, 6.11)^†^	340 (48.78)	357 (51.22)	5.087	0.024
3a/b	5.86 (5.34, 6.46)^‡^	69 (69.00)	31 (31.00)	11.234	0.001
6a/*n*	5.83 (5.36, 6.43)	26 (72.22)	10 (27.78)	5.601	0.018
Mixed	6.51 (5.55, 6.67)	4 (66.67)	2 (33.33)	0.076	0.783
Z/*χ*^2^	199.892	19.848			
*p* - value	0.001	0.001			

HCV: hepatitis C virus; IQR: interquartile range; mixed: mixed infections with two genotypes or subtypes. ^†^*p* < 0.05 versus genotype 1b; ^‡^*p* < 0.05 versus genotype 2a.

**Table 4:** Clinical and virological characteristics of chronic hepatitis C patients with DAAs treatment.

Patients' characteristics	*n* = 405
Female, *n* (%)	232 (57.14%)
Median age (IQR), years	56 (49, 63)
Median BMI (IQR), kg/m^2^	24.17 (21.97, 26.30)
Cirrhosis, *n* (%)	164 (40.39%)
Compensated	107 (26.35%)
Decompensated	57 (14.04%)
HCC, *n* (%)	12 (2.96%)
Comorbidity, *n* (%)	
Hypertension	59 (14.53%)
Type 2 diabetes	71 (17.49%)
Hematological system disease	3 (0.74%)
Median baseline HCV RNA (IQR), lg·IU/ml	6.23 (5.52, 6.71)
Noncirrhosis	6.24 (5.52, 6.76)
Compensated cirrhosis	6.26 (5.53, 6.62)
Decompensated cirrhosis	5.86 (5.12, 6.49)
Genotypes, *n* (%)	
1b	321 (79.06%)
2a	74 (18.23%)
3a	3 (0.74%)
3b	3 (0.74%)
6a	5 (1.23%)
Treatment history, *n* (%)	
Naive	379 (93.35%)
Experience	27 (6.65%)

HCV: hepatitis C virus; DAA: directing antiviral agents; BMI: body mass index; HCC: hepatocellular carcinoma; and IQR: interquartile range.

**Table 5:** Treatment response of different DAA regimens from 2018 to 2021.

Duration	DAA regimens	Response rate (CHC)	Response rate (LC)
2017–2019	OBV/PTV/r/DSV	100% (92/92)	97.22% (35/36)
	SOF + RBV	100% (41/41)	85.19% (23/27)
	EBR/GZR	100% (45/45)	100% (17/17)
	SOF + DCV ± RBV	100% (8/8)	100% (20/20)
	ASV + DCV	100% (2/2)	100% (3/3)
	GLE/PIB	100% (4/4)	100% (1/1)

2020–2021	SOF/vel ± rbv	100% (27/27)	100% (41/41)
	SOF/ldv ± rbv	100% (19/19)	100% (20/20)

DAA, directing antiviral agents; CHC, chronic hepatitis C; LC, liver cirrhosis; OBV/PTV/r, ombitasvir/paritaprevir/ritonavir; DSV, dasabuvir; SOF, sofosbuvir; RBV, ribavirin; EBR/GZR, elbasvir/grazoprevir; DCV, daclatasvir; ASV, asunaprevir; GLE/PIB: glecaprevir/pibrentasvir; VEL, velpatasvir; LDV, ledipasvir.

## Data Availability

The datasets used during the present study are available from the corresponding author upon reasonable request.
